# Cytonemes Versus Neutrophil Extracellular Traps in the Fight of Neutrophils with Microbes

**DOI:** 10.3390/ijms21020586

**Published:** 2020-01-16

**Authors:** Svetlana I. Galkina, Natalia V. Fedorova, Ekaterina A. Golenkina, Vladimir I. Stadnichuk, Galina F. Sud’ina

**Affiliations:** 1 Lomonosov Moscow State University, A. N. Belozersky Institute of Physico-Chemical Biology, 119991 Moscow, Russia; fedorova@genebee.msu.ru (N.V.F.); golyesha@mail.ru (E.A.G.); 2 Physical Department, Lomonosov Moscow State University, 119234 Moscow, Russia; stadn@polly.phys.msu.ru

**Keywords:** neutrophil, cytoneme, membrane tubulovesicular extensions, neutrophil extracellular traps (NETs), nitric oxide, actin depolymerizing microbial alkaloids

## Abstract

Neutrophils can phagocytose microorganisms and destroy them intracellularly using special bactericides located in intracellular granules. Recent evidence suggests that neutrophils can catch and kill pathogens extracellularly using the same bactericidal agents. For this, live neutrophils create a cytoneme network, and dead neutrophils provide chromatin and proteins to form neutrophil extracellular traps (NETs). Cytonemes are filamentous tubulovesicular secretory protrusions of living neutrophils with intact nuclei. Granular bactericides are localized in membrane vesicles and tubules of which cytonemes are composed. NETs are strands of decondensed DNA associated with histones released by died neutrophils. In NETs, bactericidal neutrophilic agents are adsorbed onto DNA strands and are not covered with a membrane. Cytonemes and NETs occupy different places in protecting the body against infections. Cytonemes can develop within a few minutes at the site of infection through the action of nitric oxide or actin-depolymerizing alkaloids of invading microbes. The formation of NET in vitro occurs due to chromatin decondensation resulting from prolonged activation of neutrophils with PMA (phorbol 12-myristate 13-acetate) or other stimuli, or in vivo due to citrullination of histones with peptidylarginine deiminase 4. In addition to antibacterial activity, cytonemes are involved in cell adhesion and communications. NETs play a role in autoimmunity and thrombosis.

## 1. Introduction

**Figure 1:**
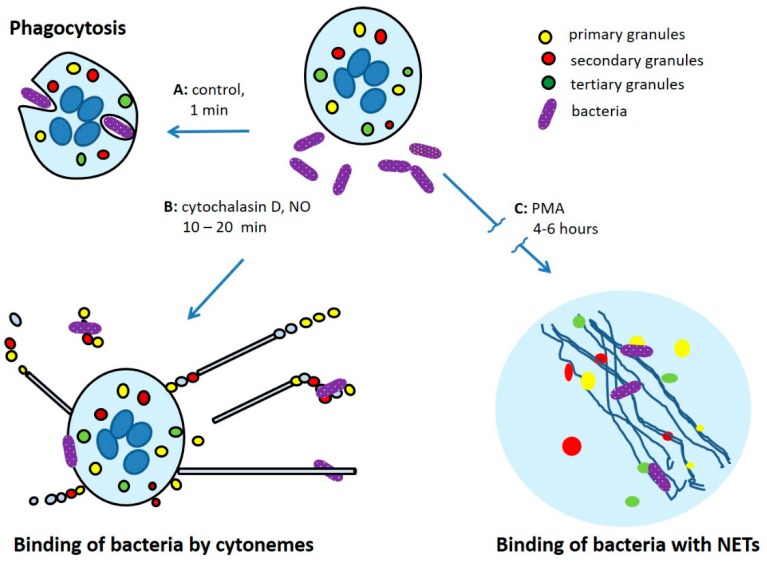
Mechanisms of interactions of human neutrophils with bacteria: (**A**) phagocytosis; (**B**) extracellular binding of bacteria by cytonemes of living neutrophils; (**C**) binding of bacteria by neutrophil extracellular traps (NETs) composed of nuclear DNA and granular proteins released by dead neutrophils.

Neutrophils belong to the innate immunity system and play an important role in protecting the body against bacterial and fungal infections. In the process of maturation of neutrophils in the bone marrow, a number of bactericidal agents are synthesized in the cells. In mature neutrophils, these agents are localized in intracellular granules of three types and secretory vesicles [1,2,3]. Neutrophils have the ability to migrate from the bloodstream to the source of inflammation, where they phagocytose and destroy bacteria with bactericidal agents, which are released from the granules into the phagosome and to the outside. Bactericidal agents act in cooperation with reactive oxygen species (ROS) formed by the NADPH oxidase complex, which is assembled on the membranes of activated neutrophils. Along with phagocytosis, neutrophils are able to bind and kill pathogens extracellularly: living neutrophils can catch microbes by a network of cytonemes while dead neutrophils scavenge pathogens with NETs, neutrophil extracellular traps (Figure 1).

Live neutrophils with intact nuclei can form on their surface multiple very long and dynamic membrane tubulovesicular extensions—cytonemes (cytoplasmic threads) [4]. Cytonemes appear to be the secretory protrusions of neutrophils that contain bactericides of primary and secondary secretory granules [5]. The formation of cytonemes in neutrophils may be initiated by intercellular mediator nitric oxide (NO) [6,7], by actin-depolymerizing microbial alkaloids such as cytochalasin D or staurosporine [4,5,8,9], by ligands of the A3-adenosine receptor [10]. The bacteria-tethering cytoneme-like protrusions were obtained also in other mammalian cells such as intestinal epithelial [11,12,13] or mast cells [14]. Mammalian cells, protozoan parasites, and bacteria widely use cytoneme-like membrane tubulovesicular secretory structures for distant cell adhesion and communications. When neutrophils use cytonemes for direct transport of their antimicrobial agents to pathogens, pathogens use their own cytoneme-like protrusions to transport their virulence factors and toxins over distance [15,16,17,18].

Dead neutrophils also can contribute to the host defense against infections releasing chromatin and proteins for the building of so called neutrophil extracellular traps (NETs). According to authors of the initial papers, NETs consist of filaments of decondensed nuclear DNA associated with histones and granular bactericidal agents. NETs possess the antibacterial activity that depends on the integrity of the DNA, since it can be eliminated with DNase [19,20]. Initial articles have aroused great interest in the study of NETs. Due to the intensive investigations various extracellular structures containing the DNA and granular proteins of destroyed neutrophils and other myeloid cells were obtained. These structures often strongly differ in morphology and size and were formed under the influence of various factors and during different periods of time [21,22,23,24,25,26]. However, authors prefer to use the term NETs to describe all kinds of the neutrophil extracellular structures.

In this review, we would like to highlight cytonemes, which can be called “living neutrophil extracellular traps”, and emphasize their difference from NET. Despite the formal similarity in the capacity to bind and kill microorganisms outside the bodies of neutrophils, cytonemes, and NETs differ fundamentally in origin, size, and composition, as well as in their physiological roles.

## 2. Structure, Composition, and Size of Cytonemes

**Figure 2:**
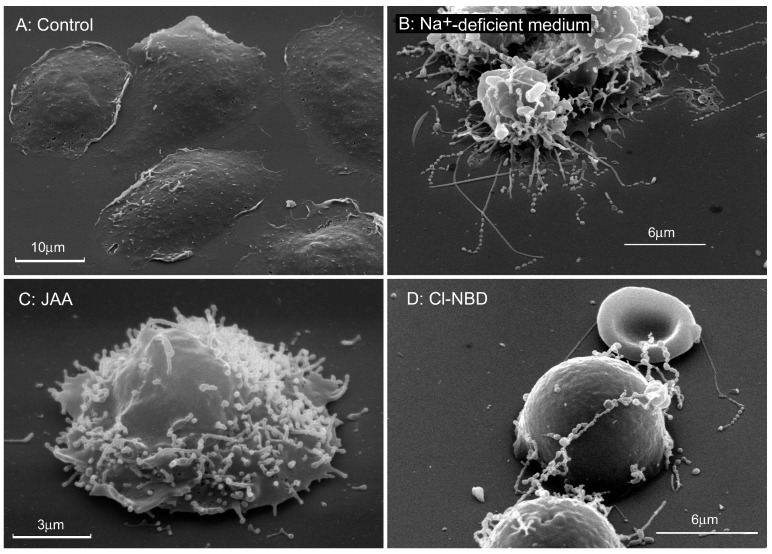
Cytonemes developed on the surface of human neutrophils in the presence of metabolic inhibitors. Scanning electron microscopy images of neutrophils that were adhered to fibronectin-coated substrata during 20 min at 37 °C: (**A**) under control conditions; (**B**) in the medium where Na^+^ ions were substituted by K^+^ ions; (**C**) in the presence of 1 mM of iodoacetic acid (IAA), inhibitor of glycolysis; (**D**) in the presence of 100 μM 7-chloro-4-nitrobenz-2-oxa-1,3-diazole (Cl-NBD), vacuolar-ATPase inhibitor. The photographs shown in this figure are similar to the photographs published in our previous article [28].

To describe tubulovesicular extensions of living neutrophils we used the term “cytonemes” that was offered for thin and long filopodia of embryonic cells [27]. This name emphasizes that filopodia contain the cytoplasm and indicate their flexible and dynamic thread-like nature (neme in Greek-thread). Cytonemes (membrane tethers, tubulovesicular extensions, nanotubes) develop during 10–20 min on the surface of living neutrophils with intact nucleus [4]. Scanning electron microscopy revealed that they consist of interconnected membrane tubules and vesicles of the same diameter that are aligned in a row (Figure 2). Cytonemes have a uniform diameter along their entire length that varies from 150 to 250 nm depending on conditions. They can reach 80–100 μm in length during 10–20 min, thereby exceeding the diameter of neutrophils more than 10 times.

Electron microscopy is the most suitable technique for studying so small structures and its interactions with bacteria, but the application of these methods is associated with considerable difficulties. The main problem is to save cytonemes during preparation for electron microscopy. Cytonemes are very vulnerable membrane structures that undergo swelling and lysis. It should also be understood that cytonemes are relatively short-lived structures. They are formed within 10–20 min and can almost immediately begin to break down as a result of the separation and lysis of vesicles at the end of the cytoneme or as a result of the shedding of cytonemes from the surface of neutrophils.

Proteome analysis revealed that cytonemes contain the bactericides of primary (myeloperoxidase, cathepsin G, and defensins) and secondary (lactoferrin, lipocalin) secretory granules of neutrophils and a number of cytosolic proteins. Cytosolic proteins include: (i) energy metabolism enzymes such as a number of glycolytic enzymes and transketolase and glucose-6-phosphate dehydrogenase; (ii) actin cytoskeleton proteins beta and/or gamma actin, L-plastin and moesin; (iii) S100 proteins and annexin 1 [5,9,29].

## 3. Factors that Affect Cytonemes Formation

The formation of cytoneme was studied mainly in the process of adhesion of human neutrophils to fibronectin-coated substrates. Cytonemes develop on the surface of neutrophils within 10–20 min. Our experimental data have revealed numerous drugs that cause the formation of cytoneme in neutrophils. These agents can be divided into groups: (1) nitric oxide donor diethylamine NONOate [6,7]; (2) microbial alkaloids disrupting actin cytoskeleton, such as cytochalasin D, latrunculin A, staurosporine [4,8,30]; (3) dynasore, an inhibitor of GTPase dynamin [9]; (4) inhibitors of glucose uptake and glycolysis [28]; (5) inhibitors of vacuolar-type ATPase (V-ATPase) [4,28]; (6) inhibitors of Cl channels and Na^+^-deficient extracellular medium [4,28]. With all the diversity of these agents, we suggest the existence of a close relationship between them. We assume that cytoneme formation in vivo occurs at the site of infection penetration. Nitric oxide (NO), which excessive production occurs in the foci of infection [31], and actin-depolymerizing alkaloids of invading microbes could be the natural initiators of the cytoneme formation. Other agents can initiate the formation of cytonemes by acting through the intracellular points of application of NO and actin-depolymerizing microbial alkaloids.

Neutrophil activation and the formation of reactive oxygen species (ROS) are not required but impair the cytoneme formation. Commonly used ROS activators, such as LPS, fMLP, and PMA, did not initiate the formation of cytonemes in neutrophils [30]. Cytochalasin D is able to cause the formation of cytonemes in both resting and activated cells, but in activated cells, cytonemes underwent fast destruction. Neutrophil activation with PMA or other stimuli leads to the production of superoxide anion radicals (O_2_^−^) formed by the NADPH oxidase complex of neutrophils. The NO radical quickly interacts with the O_2_^−^ radical, which leads to the formation of ONOO^−^ (peroxynitrite) anions [32]. This reaction reduces the bioavailabily of NO, which plays the key role in the cytoneme formation. Moreover, the produced peroxynitrite, reactive oxygen form, may initiate oxidative processes responsible for the destruction of surrounding cells and tissues [31], including oxidative destruction of cytonemes.

The importance of host-produced NO in the fight against bacteria is evidenced by the fact that many pathogenic bacteria possess NO detoxification mechanisms, such as the nitric oxide reductase (NorB) of *Neisseria meningitidis* and the flavohemoglobins (Hmp) of *Salmonella enterica* and *Escherichia* *coli* [33,34]. Bacteria of mutant lines that have lost these enzymes lose their virulence. NO, a small size unchanged molecule with unpaired electron, appear to be the ideal intercellular mediator due to its capacity to easily penetrate biological membranes [35]. This gaseous molecule produced by neutrophils themselves [36,37], macrophages, endothelium [38], and neighboring tissues exhibits broad-spectrum antimicrobial activity. NO contributes to innate host defense against *Salmonella* infections [39,40,41,42], *Campylobacter jejuni* [43], *Staphylococcus aureus* [44] and other bacteria. NO induces both nitrosative and oxidative stress that results in numerous toxic effects on bacteria [45,46]. Host NO disrupts also microbial cell-to-cell communication and suppresses staphylococcal virulence by targeting the Agr quorum sensing system [44] and disrupts zinc homeostasis in *Salmonella enterica Serovar typhimurium* [42].

**Figure 3:**
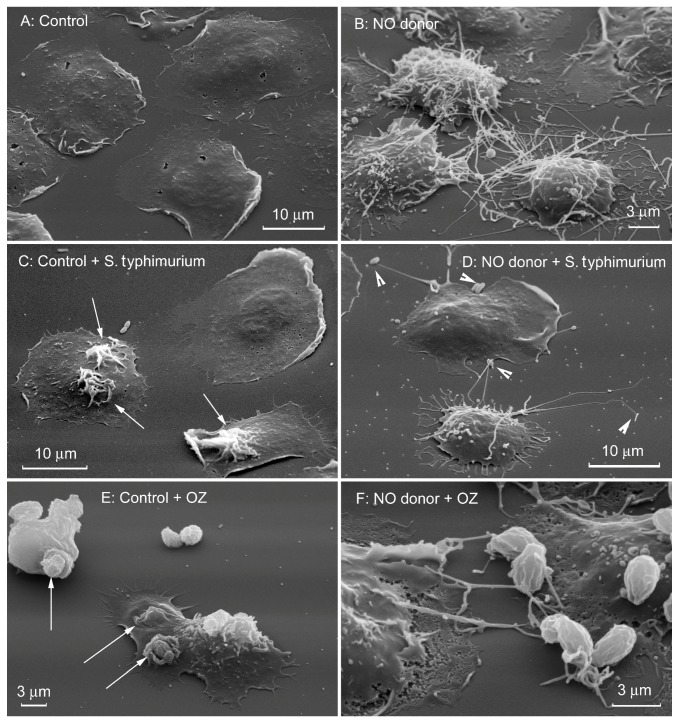
Nitric oxide (NO) shifts interactions of neutrophils with bacteria and yeast from phagocytosis to binding by cytonemes. Scanning electron microscopy images of human neutrophils plated to fibronectin-coated substrata during 20 min at 37 °C at the control conditions (**A**) or in the presence of 1 mM NO donor diethylamine NONOate (**B**). (**C**,**D**) Serum-opsonized *S. typhimurium* were added for 5 min at 37 °C to attached neutrophils. White arrows (**C**) indicate ruffles on the cell surface considered to be the sites of bacteria entering into the cells as a result of phagocytosis. (**E**,**F**) Yeast (particles of serum-opsonized zymosan, OZ) was added for 5 min at 37 °C to attached neutrophils. White arrows (**E**) indicate specific phagocytic “cups” on the surface of control cells. In the presence of NO donor bacteria (**D**) and yeast particles (**F**) were bound by cytonemes of neutrophils. The photographs shown in this figure are similar to the photographs published in our previous articles [6,7].

We suggest that the spectrum of antibacterial activity of NO also includes the ability of this natural agent to induce the formation of cytonemes and shift the interaction of neutrophils with bacterial and fungal pathogens from phagocytosis to extracellular binding of microbes by cytonemes (Figure 3) [6,7,47].

Cytonemes, apparently, are modified secretory protrusions of neutrophils, since they contain bactericides of primary and secondary secretory granules [5]. How NO turns the secretory process of neutrophils into cytoneme formation remains unknown. However, numerous data indicate the ability of NO to suppress late stages of exocytosis (the granule emptying) in chromaffin cells [48], to inhibit exocytosis of Weibel–Palade bodies in endothelial cells [49], to block exocytosis of granules in platelets [50] and killer cells [51,52].

Inhibition of oxidative phosphorylation or inhibition of P- and F-type ATPases did not cause cytoneme formation. In contrast, inhibition of the metabolism of glucose or vacuolar-type ATPase (V-ATPase) caused the appearance of membrane tubulovesicular extensions (cytonemes) on the surface of neutrophils [28]. The assembly of V-ATPase and its proton pump activity are closely related to glucose metabolism and glycolytic enzymes [53,54,55]. Data from our previous study [7,28] suggest that NO could initiate the formation of cytoneme via inhibition of glycolysis and/or V-type ATPase. It is shown that the target points of NO inside the cells is the key glycolytic enzyme glyceraldehyde-3-phosphate dehydrogenase (GAPDH) [56,57]. On the one hand, NO inhibits the enzyme by modifying the thiol groups of GAPDH by S-nitrosylation [56]. On the other hand, NO enhances the binding of NAD, the GAPDH cofactor, to GAPDH, thus inhibiting its activity [57]. The experimental data also indicate that NO inhibits V-ATPase through the formation of a disulfide bond between cysteine residues at the catalytic site and that NO can act as a negative regulator of V-ATPase activity in vivo [58].

The role of GAPDH and V-ATPase in the formation of cytonemes may be associated with their participation in membrane fusion/fission events. Numerous studies show that GAPDH has fusogenic activity against membrane bilayers, intracellular granules and liposomes [59,60,61,62,63]. The V-ATPase, which pumps protons out of cytoplasm, consists of an ATP binding domain (V1) and an integral domain (V0) that forms a proton pore in the membrane. It is suggested that membrane fusion requires the physical presence of the V0 sector of the V-ATPase [64,65,66,67,68]. Membrane fission, in contrast, depends on proton translocation activity of the V-ATPase [69]. Inhibition of GAPDH and or V-ATPase can interfere with membrane fusion/fission processes necessary for the formation of exocytotic carriers and their separation from the plasma membrane. As a result, exocytotic traffic extends from the cells in the form of tubulovesicular membrane extensions-cytonemes. The ability of Cl^−^ channel inhibitors and Na^+^-deficient extracellular medium to induce the formation of cytoneme in neutrophils can also be mediated by inhibition of V-ATPase activity through violation of charge compensation during translocation of protons by the V-ATPase.

Another target for NO within cells is the actin cytoskeleton. It is shown that (beta/gamma) actin present in human neutrophils is a substrate for NO-dependent ADP ribosylation and that this modification is associated with inhibition of actin polymerization [70].

## 4. How Cytonemes Scavenge Microbes: Advantages Over Phagocytosis

**Figure 4:**
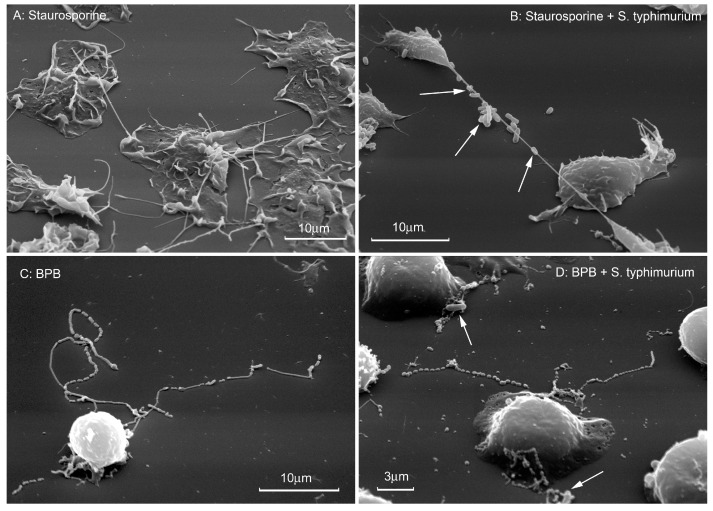
Binding of bacteria by cytonemes formed by agents acting on the actin cytoskeleton. Scanning electron microscopy images of human neutrophils that were attached to fibronectin-coated substrata in the presence of 200 nM staurosporine (**A**,**B**) or 20 µM 4-bromophenacyl bromide (BPB) (**C**,**D**). Bacteria *S. typhimurium* were added to attached neutrophils (bacteria/cells ratio 20:1) for 5 min at 37 °C. Then cells were fixed for electron microscopy. White arrows indicate bacteria bound by cytonemes. The photographs shown in this figure are similar to the photographs published in our previous articles [5,7,8].

Binding of bacteria by cytonemes does not lead to next phagocytosis of microbes, but rather causes shedding of cytonemes with bound bacteria from the cell surface. The destruction of bacteria can occur due to the release of bactericidal agents from the membrane vesicles and tubules that make up cytonemes [29]. Extracellular binding of microbes by cytonemes has several advantages compared with phagocytosis. In order for a neutrophil to phagocytose a microorganism, the latter must be located on the surface of the phagocyte. Cytonemes, the length of which often reaches ten diameters of neutrophils, increase the volume in which bacteria are available for neutrophils, hundreds of times (Figure 4). Cytonemes can deliver neutrophil bactericides without dilution and specifically to the bacteria associated with them. Finally, cytoneme-associated bacteria do not enter cells, where bacteria can survive and cause chronic infections [29].

Aggressive neutrophil bactericidal proteases and pore-forming peptides in cytonemes are packed into membrane vesicles and tubules [5]. In one hand membrane-packed aggressive bactericidal agents of neutrophils cannot damage the surrounding tissues. On the other hand, bactericidal proteases of neutrophils are protected against inactivation with serum and tissue protease inhibitors such as serpins and TIMPs and are not diluted in the extracellular medium.

## 5. The Role of Microbial Alkaloids Depolymerizing Actin in the Formation of Cytonemes

It has been shown that many invading bacterial or fungal pathogens secrete alkaloids that destroy the actin cytoskeleton to meet the needs of bacteria. Microbial alkaloids that cause actin depolymerization, such as cytochalasin D, effectively inhibit phagocytosis. But actin depolymerization simultaneously creates an alternative way for neutrophils to kill bacteria. The destruction of actin filaments serves as a signal for triggering exocytosis in neutrophils and other cells [71,72,73]. We suggest that actin depolymerization at the same time modifies the exocytotic process, as will be described later. As a result, secretory traffic emerges from the cells in the form of growing cytonemes, filiform membrane tubulovesicular structures containing bactericidal agents typical of primary and secondary secretory granules and capable of binding microbes [4,5,8].

Cytoneme formation in neutrophils can be initiated by various ways of influencing the actin cytoskeleton. Cytochalasin D inhibits actin polymerization via binding to the barbed end of actin filaments [74]. Staurosporine, the natural alkaloid of *Streptomyces staurosporeus,* acts via inhibition of cofilin phosphorylation. Cofilin, the actin-severing protein, is activated upon dephosphorylation at serine 3, which results in a breakdown of the filamentous actin [75]. The alkylating agent 4-bromophenacyl bromide (BPB), which is a reliable inducer of cytoneme formation, affects actin cytoskeleton through a leukocyte-specific actin-bundling protein, L-plastin [76].

Cytonemes contain actin and other proteins involved in the polymerization of actin, as shown in numerous works and collected in scientific reviews [77,78,79]. Speaking about the actin-depolymerization-induced formation of cytonemes, we contradict many authors who consider that actin polymerization is the driving force that pushes cytonemes out of the cell. Cytonemes with fluorescent labelling of actin are visualized in the form of thin and long linear structures, suggesting the presence of long actin filaments. However, cytoneme, which have 150–250 nm in diameter, filled with fluorescently-labeled monomeric actin look exactly the same when studied using fluorescence (visible) microscopy. In addition, it is difficult to imagine how actin polymerization can drive the extension of cytonemes partially or completely consisting of a row of vesicles.

Another argument in favor of the actin-driven nature of cytonemes is the partial suppression of cytoneme formation by cytochalasin D, for example, in experiments with neutrophils [10]. The formation of cytonemes in neutrophils was evaluated after incubation of cells with cytochalasin D for an hour, as is usually the case with fibroblasts and other cells. However, the authors did not take into account that many cellular processes in neutrophils proceed faster. The formation of cytonemes under the action of cytochalasin D occurs within 10–30 min, and an hour after the addition of this agent, significant destruction of cytonemes is observed as a result of swelling, lysis and shedding of these structures. Therefore, the authors could observe not blocking the formation, but the partial destruction of cytonemes stimulated by prolonged incubation of neutrophils with cytochalasin D. In connection with the foregoing, we believe that it is more correct to call cytonemes actin-containing, but not actin-driven structures.

Actin depolymerization may cause the cytoneme formation in neutrophils via stimulation of NO production due to activation of NO synthase (NOS). Actin presents in the cells as F-actin (polymer filamentous actin) and G-actin (monomer or globular actin). As was shown for platelets and endothelial cells G-actin, resulting from the depolymerization of filamentous actin, but not F-actin, can directly associate with nitric oxide synthase, thereby stimulating the production of NO [80,81,82,83,84].

The actin cytoskeleton may play a coordinating role in the complex interaction of glycolytic enzymes and vacuolar-type ATPases in the implementation of membrane fusion/fission processes required for the formation and separation of exocytotic transport carriers from the plasma membrane. This assumption is supported by the ability of glycolytic enzymes aldolase, GAPDH or phosphoglucoisomerase at the protein level to interact with actin filaments [85,86]. The protein subunits B and C belonging to the V1 domain of the V-ATPase also contain binding sites for filamentous actin [87,88,89].

**Figure 5:**
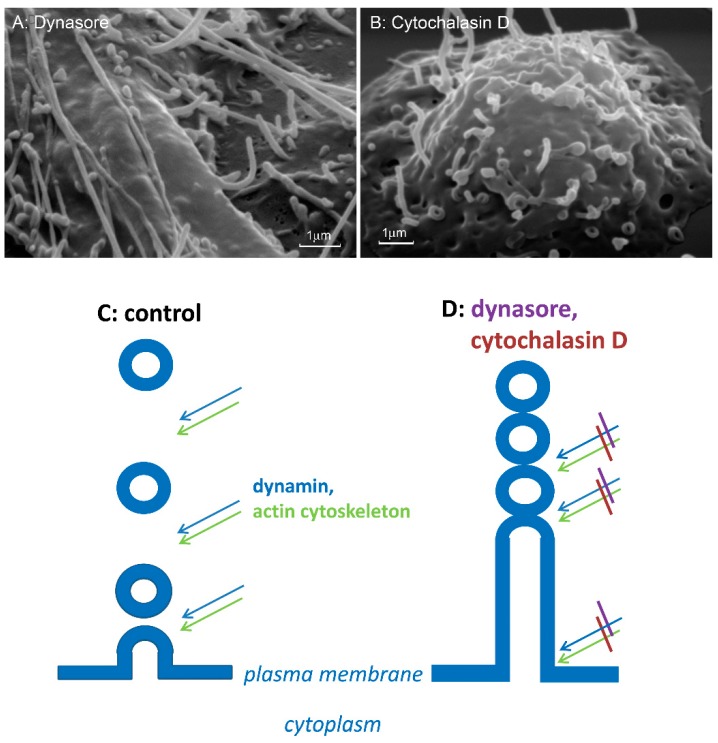
Scheme of the cytoneme formation. (**A**,**B**) Scanning electron microscopy images of the surface of neutrophils that were attached to fibronectin-coated substrata during 20 min in the presence of 200 µm dynasore (**A**) or 10 µm cytochalasin D (**B**). (**C**,**D**) GTPase dynamin, in collaboration with an intact actin cytoskeleton separates exocytotic vesicles from the plasma membrane (**C**). Inhibition of dynamin with dynasore and/or depolymerization of actin filaments with cytochalasin D block the cleavage of exocytotic vesicle from the plasma membrane and from each other, and the secretory process extends from the cells as tubulovesicular cytonemes (**D**). The photographs shown in this figure are similar to the photographs published in our previous article [9].

Finally, actin cytoskeleton depolymerization can initiate cytoneme formation by inhibiting the separation of exocytotic vesicles from the plasma membrane (Figure 5). Exocytosis in neutrophils can occur via different mechanisms, including budding of a variety of membrane vesicles (ectosomes, microvesicles) [90,91,92,93,94]. Among them there are vesicles with direct antibacterial activity that are able to bind to bacteria and kill them [93,95]. We suggest that budding of microvesicles with antibacterial activity and the formation of cytonemes are modifications of the same cell secretion process. The mechanism of separation of secretory vesicular and tubular carriers from the plasma membrane has not been studied. During endocytosis a GTPase dynamin in cooperation with intact actin cytoskeleton executes the fission of endocytic membrane vesicles from the plasma membrane [96,97,98]. Dynamin assembles into higher order oligomers at the neck of budding vesicles, binds to GTP and undergoes a nucleotide-dependent conformational change that leads to vesicle fission from the initial membrane. Actin filaments appear to generate the force required for vesicle separation [99,100]. Our data revealed that specific inhibitor of GTPase dynamin dynasore and actin depolymerizing agent cytochalasin D initiated the formation the cytonemes which are similar in size and composition with those in neutrophils (Figure 5A,B). By analogy with the process of endocytosis, we assume that inhibition of dynamin or depolymerization of actin blocks the separation of exocytotic vesicles from the plasma membrane of neutrophils and from each other. As a result, the secretory process emerges from the cells in the form of tubulovesicular cytonemes [9].

## 6. Cytonemes in Embryonic, Blood and Other Eukaryotic Cells

Cytoneme formation is not a specific phenomenon for neutrophils. The formation of cytonemes was first observed in embryonic cells of a sea urchin during gastrulation. Modern research considers cytonemes as signal structures providing a distance relationship between structures formed in embryos [27,77,101]. In intestinal epithelial cells, *Clostridium difficile* toxin, which causes the depolymerization of the actin cytoskeleton through ADP-ribosylation [102,103], causes the formation of *Clostridia*-tethering cytoneme-like protrusions [11,12]. Breast carcinoma cells suspended above weakly adhesive substrates produce cytoneme-like long and dynamic membrane tubular structures, the length of which increased significantly under the influence of cytochalasin D or latranculin A. Since the formation of blood-borne metastases depends on the interaction of cells with substrates and other cells, long sticky membrane structures in unattached transformed epithelial cells of the breast can play an important role in the spread of breast cancer metastases [104,105]. In primary spleen lymphocytes and Bal 17 cells, the binding of the B cell antigen receptor by immunoglobulin M (IgM), an antigen surrogate, led to the appearance of very long threadlike structures [106]. It is assumed that these flexible and dynamic filopodia play an important role in the distance interaction between B cells stimulated by antigen and other immune cells in the formation of the immune response. Similar tubulovesicular extensions, a ‘beads-on-a-string’ structures or apoptopodia were observed in THP-1 cells and primary human CD14 monocytes undergoing ultraviolet-induced or spontaneous apoptosis. These beaded apoptopodia were involved in process of generation of apoptotic bodies. Cytochalasin D in combination with trovafloxacin12, caspase-activated pannexin 1 channel inhibitor, stimulated the formation of such tubulovesicular apoptopodia [107].

Transfection of B144/LST1, a gene in the tumor necrosis factor cluster, can cause development of dynamic cytoneme-like long filopodia containing actin in a variety of eukaryotic cells. B144/LST1 gene encoded in human major histocompatibility complex is highly expressed in professional antigen-presenting dendritic cells. The occurrence of dynamic cellular extensions reaching in length several cell diameters offers a possible mean for finding the T cell whose receptor structure is present to recognize the antigen presented by the dendritic cell [108].

## 7. Cytonemes in Cell Adhesion and Communication of Parasitic Protozoa and Bacteria

Cytoneme-like secretory structures play an important role in cell adhesion and communication of parasitic protozoa. Filamentous protrusions of the plasma membrane 200 nm wide are formed within a few minutes after the activation of the gametocyte of the malaria parasite *Plasmodium falciparum* in the mosquito’s midgut. The projections have a beaded structure and contain the adhesive gamete proteins Pfs230, Pfs48/45, or Pfs25. They establish long-term intercellular connections between the sexual stages of parasites [17]. Similar membrane structures were formed on the surface of the pathogen of cattle *Theileria annulata*, another member of the Apicomplexa type, in the schizont stage [109]. Authors using cryo-electron tomography detected thin actin filaments beneath these protrusions, and considered that the extension is driven by schizont actin polymerization. It should be noted that a characteristic feature of protozoan parasites of the Apicomplexa type is the practical absence of filamentous actin in vivo. Actin is present in parasites mainly in monomeric form. Actin filaments isolated from parasite lysates or formed in vitro were very short and did not exceed 150 nm in length [110]. The filaments that the authors observed in parasites did not exceed this length and were oriented in different directions. It is unlikely that the actin polymerization in this case can be the cause of the extension of structures reaching 10 µm in length. The interpretation of these structures as actin-containing but not actin-driven in this case seems more correct. African *Trypanosoma brucei*, a pathogen responsible for human sleeping sickness, produces membrane tubules that originate from the flagellar membrane and dissociate into free extracellular vesicles [15]. These vesicles contain proteins that contribute to the virulence of the parasites and cause remodeling of the red blood cells of the host, causing anemia. 

**Figure 6:**
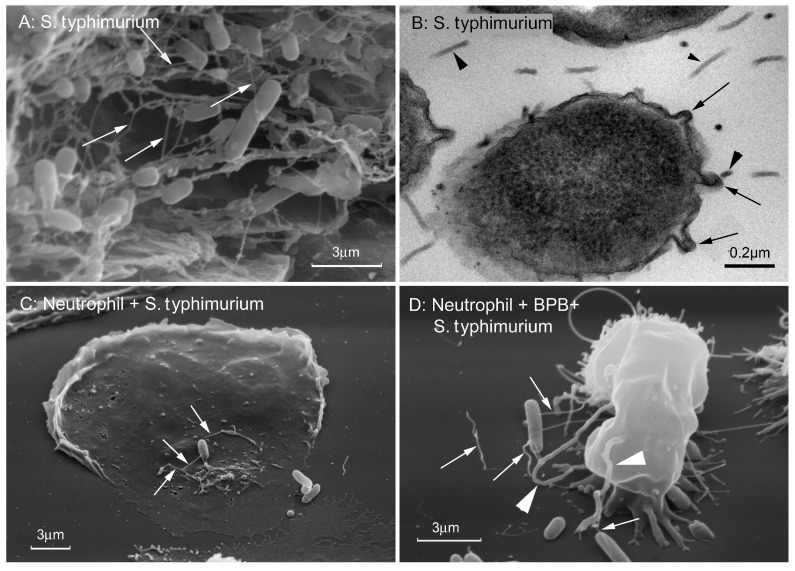
Cytonemes of *Salmonella typhimurium*. (**A**) Scanning electron microscopy images of *S. typhimurium* biofilm grown on gallstones. White arrows indicate 60 nm wide cytonemes interconnecting bacteria in biofilm. (**B**) Transmission electron microscopy images of a thin section of *S. typhimurium* biofilm grown on agar. Black arrows indicate fragments of 60 nm-wide outer membrane tubular extensions (cytonemes) of bacteria. Black arrowheads indicate 25 nm in diameter bacterial flagella. (**C**,**D**) Scanning electron microscopy images of *S. typhimurium* attached to the control (**C**) and 4-bromophenacyl bromide (BPB)-treated (**D**) neutrophils. White arrows indicate the 60 nm-wide bacterial cytonemes. White arrowheads indicate the cytonemes of neutrophils with a diameter of 200 nm. The photographs shown in this figure are similar to the photographs published in our previous article [18].

Gram-negative bacteria remotely interact with other bacteria, eukaryotic cells and substrates using cytoneme-like protrusions of the outer membrane of bacteria (Figure 6).

Early electron microscopic study of the flagella of *Vibrio metchnikovii*, *Bdellovibrio bacteriovorus*, or *Helicobacter pylori* revealed an internal electron-dense filament and the surrounding membrane sheaths of the flagellar [111,112,113]. These membrane sheaths were often beaded to a variable degree and often did not contain flagella filaments [114]. Outer membrane tubular appendages interconnect bacteria *Salmonella typhimurium* in biofilms and attach bacteria to eukaryotic cells and substrates [18,115,116]. The diameter of the appendages (60 nm) significantly exceeded the diameter of the bacterial flagella (15–20 nm) or pili (6–7 nm). *Francisella novicida*, the causative agent of tularaemia, constitutively releases spherical and tubular outer membrane vesicles that function to deliver virulence factors to host cells. A study of whole bacteria revealed the presence of tubes extending out from the outer membrane of the bacterial cell wall [16]. Similar outer membrane structures with tubes and chain-like structures were observed in Gram-negative bacteria such as *Myxococcus xanthus*, a rod-shaped species found in the upper soil layer [117,118], hyperthermophilic archaea *Thermococcus* [119] or metal-reducing microbe *Shewanella oneidensis* MR-1 [120,121].

## 8. Structure, Size, and Composition of NETs

The term “neutrophil extracellular traps” (NETs) was proposed by Arturo Zychlinsky and his colleagues for structures formed by chromatin and proteins released by neutrophils that died as a result of 4–6 h activation with PMA (phorbol 12-miristate 13-acetate) or other stimuli. According to the authors, the NETs formed in vitro experiments consist of 15 nm in diameter strands of decondensed nuclear DNA associated with histones [19], with fibrils of NETs ten times less in diameter when compared to cytonemes. The filaments are dotted with globular structures with a diameter of about 50 nm, which contain neutrophilic granular bactericides. Bactericides of primary, secondary and tertiary granules of neutrophils are adsorbed on DNA-strands. The main components appear to be elastase and myeloperoxidase, which belong to the primary granules. Despite such a small size, NETs are mainly studied using confocal microscopy. In confocal microscopy the framework of filaments often appear as cloud-like structures that occupy 10–15 times a larger area than the neutrophil itself [19,20,21].

Electron microscopy methods are more suitable for studying NETs and their interaction with pathogens, but the correct application of these methods is complicated. The interaction of NET with bacteria occurs at a time when neutrophils are already destroyed and, accordingly, are absent in the preparations. Therefore, it should be proved that the filaments that trap bacteria consist of neutrophilic components, but are not filamentous structures of the bacteria themselves. Bacteria can quickly come into contact with each other using their own filiform structures, such as membrane tubules 60 nm wide, flagella 20–25 nm wide or pili 10–15 nm wide. It is a difficult but possible task for electron microscopy to distinguish NETs from bacterial filaments of very close diameters using antibodies labeled with colloidal gold. This method, for example, was used to distinguish between NET networks and fibrin in fibrin-purulent inflammatory lesions in humans [122].

## 9. NETs Formation In-Vitro and In-Vivo

Great interest to the study of NETs was facilitated by the simplicity of the method proposed by the authors for obtaining NETs in vitro. Isolated neutrophils are incubated 4–6 h with PMA (phorbol 12-myristate 13-acetate) or other stimuli in the plain Petri dishes [19,20]. PMA is known as a powerful inducer of the reactive oxygen species (ROS) formation in neutrophils. In vitro NETs are formed as a result of neutrophil ROS-dependent cellular death—NETosis, which is distinct from apoptosis and necrosis. Upon NETosis, the initial loss of all intracellular membranes is followed by the disintegration of the plasma membrane. The NET formation includes several sequence steps: the NADPH oxidase-generated ROS formation, which initiates the transport of neutrophil elastase, and, subsequently, myeloperoxidase from granules to the nucleus; histone modification by elastase and chromatin decondensation; and, finally, the disruption of plasma membrane and release of chromatin and the NETs formation [123,124]. Neutrophils isolated from the blood of patients with chronic granulomatous disease that carry mutations in ROS-generating NADPH oxidase cannot form NETs [20].

Lysis of neutrophils can occur under the influence of toxins produced by gram-positive bacteria *Staphylococcus aureus.* Pathogen-induced lytic neutrophil cell death is considered to be immune evasion strategy. Survival of bacteria is partially attributed to the ability of the pathogen to avoid destruction by human neutrophils, which are crucial to the host immune response to *S. aureus* infection. Upon encountering neutrophils bacteria *S. aureus* induces the production of bicomponent pore-forming toxins leukocidin and gamma hemolysin, which serves as an extra-and intracellular weapon to protect the bacterium from destruction by human neutrophils [125]. Phagocytosed *S. aureus* can escape phagolysosomal killing by attacking the neutrophil from the inside out [126]. Whether NETs formation occurs in these cases is unknown. However, in the literature such a process is often called lytic NETosis [127].

At least a process of NETs formation that did not require neutrophil lysis was described as vital NETosis [127,128]. According to the authors, in neutrophils responding to *S. aureus* the nucleus rapidly became rounded and condensed and separated membrane vesicles filled with nuclear DNA. The vesicles were extruded intact into the extracellular space where they ruptured, and the chromatin was released. This entire process occurred very rapid (5–60 min) in an oxidant-independent manner. A similar rapid neutrophil extracellular trap formation in response to *C. albicans* hyphae was found to depend on β-glucan recognition by CR3 and require fibronectin [129] In these cases, an additional study of the process of DNA secretion from the nucleus into the extracellular space using special DNA labeling suitable for electron microscopy would be especially useful.

Recent data suggest that during infection in vivo NET formation may occur by different mechanisms [23]. In contrast to in vitro experiments, in vivo studies of NET formation do not support a requirement for ROS in NET formation [130]. Neutrophilic elastase was believed to be necessary for histone cleavage during NET formation. But recently, data have been published demonstrating NET formation in elastase-deficient mice in an experimental deep vein thrombosis model [131]. It is suggested that the basis for the formation of NETs in vivo is decondensation of chromatin that is mediated by peptidyl arginine deiminase 4 (PAD4). PAD4 is activated by Ca^2+^ signal in response to physiological activators such as bacteria. PAD4 catalyzes the conversation of histone arginines to citrullines, thus reducing the positive charge of histones and weakening histone-DNA interactions [132]. Histone deimination in human neutrophils is induced in response to inflammatory stimuli, such as LPS, TNF, fMLP or hydrogen peroxide, but not by treatments that induce apoptosis [133]. It was shown that neutrophils from PAD4-deficient mice cannot form NETs after stimulation with chemokines or incubation with bacteria, and are deficient in bacterial killing by NETs [130,134]. Inhibition of PAD4 activity was shown to be sufficient to disrupt mouse and human NET formation [135].

However, not only the mechanism of formation, but also the structure of NETs that have been formed in vivo, may differ from the structure of NETs that are formed in vitro. Kolaczkowska with coauthors in experiments performed in vivo demonstrated that in bloodstream infection neutrophils infiltrate into the liver and release NETs, which were localized on the vessel walls [130]. DNase treatment removed only DNA but not histones and neutrophil elastase from the vessel walls, thus demonstrating that histones and elastase were not localized on DNA strands as in the NET model proposed by Zychlinsky′s laboratory.

## 10. How NETs Kill Microbes

Antimicrobial effect of NETs critically depends on the integrity of the DNA structure, since the action of DNase resulted in the loss of the bactericidal potential of the “traps” [19,136]. DNA possesses a rapid bactericidal activity due to its ability to sequester surface bound cations, disrupt membrane integrity and lyse bacterial cells. Direct contact and the phosphodiester backbone are required for the cation chelating, antimicrobial property of DNA. By treating NETs with excess cations or phosphatase enzyme, the antimicrobial activity of NETs is neutralized, but NET structure, including the localization and function of NET-bound proteins, is maintained [135]. Citrullination of histones, which weaken histone-DNA interactions, lead to decondensaiton of DNA and make it possible to direct interactions of DNA with bacteria. The bactericide function of NETs might be also due to histones, which also possess antimicrobial activity [24,137].

The contribution of neutrophilic granular bactericidal agents, which are absorbed onto negatively charged DNA strands and uncovered by membrane, in the NET antimicrobial activity is unclear. Small cationic proteins of neutrophils including defensins kill microbes via inserting into the negatively charged membranes of bacteria. Whether DNA-associated proteins maintain their capacity to insert into bacterial membrane remains to be elucidated [23]. Serine proteases, such as cathepsin G, elastase and protease 3, which are released upon neutrophil destruction and NET formation, can be quickly inactivated by themselves through proteolysis. The aforementioned processes can explain the experimental results when the bacteria associated with NETs, after the destruction of the NETs, turned out to be alive [138].

When NET formation occurs in vivo, proteases released by neutrophils can also be inactivated by plasma and tissue protease inhibitors, including irreversible serine protease inhibitors (serpins) and tissue matrix metalloproteinase inhibitors (TIMPs). [139,140].

In connection with the above, questions arose about the place of NETs in immune defense. NETs may be of more importance in autoimmunity and thrombosis than in innate immunity [23,26,138,141,142,143].

## 11. NETs in Thrombosis, Autoimmunity, and Inflammation

NETs can serve as a framework for thrombosis. Extracellular DNA fibers produced in inflammation stimulate thrombus formation and coagulation and are abundant in thrombi in animal models of deep vein thrombosis [144,145]. Neutrophil histone modification by PAD 4 is critical for deep vein thrombosis in mice [146,147]. Cancer patients with elevated level of citrullinated histone H3, a biomarker for NET formation, experience a higher cumulative incidence of venous thromboembolism [148]. Recently it is shown that host DNases1 and DNases1-like 3 can prevent vascular occlusion based on NETs [149].

Autoimmune diseases such as systemic lupus erythematosus or rheumatoid arthritis are characterized by the circulation of autoantibodies that recognize intracellular antigens. NETs consist of intracellular antigens such as DNA, histones and granular proteins, which are known targets of antibodies in each disease. Antibodies to citrullinated histone H4 included in NETs were observed in patients with rheumatoid arthritis or systemic lupus erythematosus [150,151]. PAD4 have emerged as potential therapeutic targets for the treatment of rheumatoid arthritis [152]. Patients with anti-neutrophil cytoplasmic antibody-associated vasculitis develop autoantibodies that recognize NET components such as proteinase 3 and myeloperoxidase [153]. The disruption of neutrophils during NETs formation in vivo is accompanied with the release to the outside of highly aggressive bactericidal agents, which cause the inflammation of surrounding cells and tissues. The NET formation releases also large amounts of histones into tissues where they can target microbes but also cause tissue damage [24]. The pro-inflammatory effect of NETs is also indicated by the fact that the suppression of the NET formation in PAD4- and elastase-deficient mice protects the host tissues from damage during Staphylococcus aureus infections [130]. The resolution of infection-associated inflammation and clearance of NETs in the body can occur through two mechanisms, such as DNase-dependent digestion and phagocytosis by macrophages [154,155].

## 12. Conclusions

In this review, we compared two mechanisms of extracellular elimination of pathogenic microbes by neutrophils, such as microbial binding by cytonemes of living neutrophils or by NETs, which are composed of components of dead neutrophils. Cytonemes are flexible and mobile chains of membrane tubes and vesicles of the same diameter, protruding from the cell body. They contain bactericides of primary and secondary secretory granules and have the ability to bind microbes and kill them with bactericides released from membrane vesicles and tubules as a result of lysis. Cytonemes many times expand the space in which pathogens will be accessible to neutrophils. Bactericides are released in the immediate vicinity of associated bacteria, without being diluted in the medium and without destroying the surrounding tissues. Cytoneme-like secretory structures play a role in the cell adhesion and communication of mammalian cells, bacteria, or protozoan parasites. Secretion with cytonemes can deliver signaling molecules at a distance exactly to the destination and without diluting. 

After the death of neutrophils as a result of NETosis, DNA and bactericidal agents of neutrophils continue to protect the body against infections in the form of NETs. Decondensed DNA strands associated with histones are the main constructive components of NETs formed in vivo and in vitro. The bactericidal activity of NET is associated with the DNA itself, histones and granular bactericidal agents absorbed onto the DNA strand. Neutrophilic aggressive components are not covered with a membrane, so they can damage the surrounding tissues, inactivate themselves and cause inflammation. Formed inside the blood vessels NETs can serve as the basis for the development of thrombosis. In addition, DNA, histones and granular proteins are intracellular antigens that can stimulate autoimmune processes.

Neutrophils are short-lived cells that provide the first line of defense against invading pathogens. They are quickly recruited to the site of infection. Proper resolution of the infection requires precise regulation of neutrophils. Phagocytosis of neutrophils by macrophages in the focus of inflammation serves to limit the spread of infection and prevent autolysis of neutrophils [156]. By phagocytosis, macrophages remove non-self (for example, invading microbes) and altered self-cells and cell debris. Classical examples of unwanted self-cells are aging neutrophils and neutrophils after interaction with microbes in injuries and infectious areas including neutrophils that undergo NETosis [153]. The phagocytosis of neutrophils by the macrophage results in the enhancement of the comparatively limited macrophage antimicrobial capacity by the acquisition and use of potent neutrophil bactericidal molecules [156]. Thus, phagocytosis of neutrophils and their remains by macrophages, the final event in the life of neutrophils, gives a new life for a huge antibacterial arsenal of neutrophils in the fight against infection.

## Abbreviations

NETs	neutrophil extracellular traps
NO	nitric oxide
PMA	phorbol 12-myristate 13-acetate
LPS	lipopolysaccharide from Salmonella enterica serovar Typhimurium
fMLP	N-formylmethionyl-leucyl-phenylalanine
TIMPs	tissue inhibitors of metalloproteinase
